# Molecular Mechanisms of Adipogenesis: The Anti-adipogenic Role of AMP-Activated Protein Kinase

**DOI:** 10.3389/fmolb.2020.00076

**Published:** 2020-05-08

**Authors:** Bilal Ahmad, Christopher J. Serpell, Isabel Lim Fong, Eng Hwa Wong

**Affiliations:** ^1^School of Biosciences, Faculty of Health and Medical Sciences, Taylor’s University, Subang Jaya, Malaysia; ^2^School of Physical Sciences, University of Kent, Canterbury, United Kingdom; ^3^Department of Paraclinical Sciences, Faculty of Medicine and Health Sciences, Universiti Malaysia Sarawak, Kota Samarahan, Malaysia; ^4^School of Medicine, Faculty of Health and Medical Sciences, Taylor’s University, Subang Jaya, Malaysia

**Keywords:** obesity, adipogenesis, WAT, BAT, AMPK, beige/brite adipocytes

## Abstract

Obesity is now a widespread disorder, and its prevalence has become a critical concern worldwide, due to its association with common co-morbidities like cancer, cardiovascular diseases and diabetes. Adipose tissue is an endocrine organ and therefore plays a critical role in the survival of an individual, but its dysfunction or excess is directly linked to obesity. The journey from multipotent mesenchymal stem cells to the formation of mature adipocytes is a well-orchestrated program which requires the expression of several genes, their transcriptional factors, and signaling intermediates from numerous pathways. Understanding all the intricacies of adipogenesis is vital if we are to counter the current epidemic of obesity because the limited understanding of these intricacies is the main barrier to the development of potent therapeutic strategies against obesity. In particular, AMP-Activated Protein Kinase (AMPK) plays a crucial role in regulating adipogenesis – it is arguably the central cellular energy regulation protein of the body. Since AMPK promotes the development of brown adipose tissue over that of white adipose tissue, special attention has been given to its role in adipose tissue development in recent years. In this review, we describe the molecular mechanisms involved in adipogenesis, the role of signaling pathways and the substantial role of activated AMPK in the inhibition of adiposity, concluding with observations which will support the development of novel chemotherapies against obesity epidemics.

## Introduction

Obesity is an increasingly prevalent disorder around the globe promoted by genetic, nutritional, and environmental factors. Energy imbalance – excessive consumption of calories compared to utilization is the key driving force of obesity. Obesity is a multifactorial chronic disease, linked to other disorders including cancer, insulin resistance, cardiovascular diseases and type-2 diabetes (Cohen et al., 2014; Tung et al., 2017; Ghaben and Scherer, 2019). Obesity/overweight is now the fifth leading cause of death worldwide (Chandrasekaran et al., 2012). According to World Health Organization (WHO), in 2016, more than 1.9 billion adults were overweight, of whom 650 million were suffereing from obesity, with approximately 2.8 million adults dying each year due to the condition (World Health Organization, 2020). There are many factors which contribute to obesity such as sedentary lifestyle, high calorific intake, depression, and various social and monetary issues, but they have a single common result: the accumulation of fats in mature adipocytes of white adipose tissue (WAT): obesity is characterized by increase in the mass of adipose tissue (Ghaben and Scherer, 2019). The epidemic of obesity has therefore focused researchers’ attention on understanding the development of adipose tissue and fat cells, regulated by a multi-step process known as adipogenesis (Lefterova and Lazar, 2009). Improved and holistic knowledge of the processes governing adipogenesis is required if we are to counter the burgeoning epidemic (Rosen and MacDougald, 2006).

Adipose tissue plays an important proper role in the body. Excess energy is stored in the form of fat, in mature adipocytes within WAT (of which these cells make up the majority) (Niemalä et al., 2008), and during energy scarcity, these fats are used by other organs of the body to meet the energy demand (Moseti et al., 2016). Obesity is diagnosed either on the amount of WAT or the number of mature white adipocytes in WAT (Park, 2014), rather than simply by body weight. The expansion of adipose depots can be driven either by the increase in the number (hyperplasia) or size (hypertrophy) of adipocytes (Ghaben and Scherer, 2019). Both hyperplasia and hypertrophy are responsible for the dysfunctionality of adipose tissue (Unamuno et al., 2018). Changes in the physiological functions of dysfunctional adipose tissue (such as abnormal secretion of adipokines, insulin resistance, and chronic inflammation) are directly linked to obesity and its related co-morbidities (Van Kruijsdijk et al., 2009). For example, inflammation in adipose tissue due to abnormal secretion of proinflammatory adipokines such as monocyte chemoattractant protein-1 (MCP-1), tumor necrosis factor-alpha (TNFα), Interleukin 6 (IL 6) etc. is a key factor in the development of type 2 diabetes (T2D), insulin resistance, cardiovascular diseases and cancer (Burhans et al., 2011; Park et al., 2014). So, therefore it is essential to understand the molecular mechanisms of adipocyte differentiation, physiology, and morphology of adipocytes in order to contribute to the overall understanding of adiposity and develop new ways to combat obesity and its associated complications. A concise knowledge of the role of genes, proteins (transcriptional factors and hormones), and signaling intermediates regulating adipogenesis is also of vital importance and all of these factors are promising targets for the discovery of novel anti-obesogenic drugs. In particular, the role of energy sensor protein of the body, adenosine monophosphate-activated protein kinase (AMPK, a negative regulator of white adipogenesis) in adipose tissue development is of central importance as we will argue in this review.

This review aims to provide a comprehensive knowledge of the key molecular factors (proteins and various signaling pathways) involved in adipocyte differentiation, and the anti-adipogenic role of AMPK and its mechanism of activation in adipose tissue. But before heading toward those details, we must survey the origin, physiology and types of adipose tissues, and the biomolecules responsible for the morphological changes during adipocyte differentiation.

## Adipose Tissue and Adipogenesis

Adipose tissue is one of the most complex organs in the human body, containing pre-adipocytes, adipocytes, immune cells, fibroblasts, pericytes, vascular smooth muscle cells, and vascular endothelial cells (Bijland et al., 2013; Coelho et al., 2013; Schling and Löffler, 2018). At the whole body level, adipose tissue is divided into visceral adipose tissue (VAT) and subcutaneous adipose tissue (SCAT) (Bijland et al., 2013). The tissue secretes various adipokines and has regulatory roles in the endocrine, immune and metabolic systems (Bijland et al., 2013; Booth et al., 2015). WAT is the hub for the synthesis and storage of triglycerides. Maintenance of systematic energy balance through storage and release of free fatty acids and via secretion of adipokines is the main function of WAT (Raajendiran et al., 2019). Whether located viscerally or subcutaneously, adipose tissue has a crucial role in the survival of an individual because it is the basic source of fatty acids for the production of heat and energy. White adipocytes or white fat cells are lipid-laden cells within WAT that acquire the ability to accumulate lipids after differentiation – the process in which the cells from a common ancestor are derived mitotically and become different from one another in morphology and function. Adipocytes are derived from multipotent mesenchymal stem cells (MSCs), which are first transformed into pre-adipocytes before undergoing secondary differentiation to become mature adipocytes. The differentiation of adipocytes is determined by the expression of genes and the function of proteins which dictate the phenotype of adipocytes (Ali et al., 2013). Hyperplasia and hypertrophy of WAT through adipogenesis (e.g., due to excessive energy intake accompanied by low energy expenditure) leads to obesity (Tang and Lane, 2012; Ali et al., 2013; Haider and Larose, 2019). The cellular process of adipogenesis involves three well-defined stages: (i) commitment of MSCs to the adipocyte lineage; (ii) mitotic clonal expansion - involving replication of DNA and duplication of cells; (iii) terminal differentiation, involving expression of genes and transcriptional factors such as CCAAT/enhancer-binding proteins (C/EBPs) family and peroxisome proliferator-activated receptor–γ (PPARγ) – and a dramatic increase in lipogenesis and induction of lipogenic genes such as acetyl CoA carboxylase (ACC), Fatty acid synthase (FAS) and adipocyte fatty acid binding protein (aP2) (Lefterova and Lazar, 2009; Khalilpourfarshbafi et al., 2018; Lazar et al., 2018). The differentiation of pre-adipocytes into mature adipocytes is also influenced by various other factors including growth factors such as insulin-like growth factor 1 (IGF-1) (Lefterova and Lazar, 2009), and insulin itself. IGF-1 is critical for the survival, proliferation and differentiation of pre-adipocytes (Garten et al., 2012), and insulin is one of the potent adipogenic hormones, inducing the transcription of various positive regulators of adipogenesis (Klemm et al., 2001). During adipogenesis, the cells lose their fibroblastic shape and become spherical, which is an indication of profound changes taking place in the extracellular matrix (ECM), and cytoskeletal components of the cells (Niemalä et al., 2008), including decreased expression of actin (Lazar et al., 2018). Alteration in the organization of actin may influence the cytoskeletal tension, something which has been shown to regulate adipogenesis *in vitro* (Schiller et al., 2013). Various components of the ECM, negatively or positively regulate the differentiation of pre-adipocytes (Sarantopoulos et al., 2018). For instance, proteolytic degradation of the ECM around pre-adipocytes by a cascade of plasminogen is essential for changes in the expression of adipogenic genes and deposition of fats (Selvarajan et al., 2001; Ali et al., 2013). Selvarajan et al. (2001) reported that events and changes (molecular and morphological) which were associated with these changes in ECM might modulate adipogenesis directly because they alter the expression of positive transcriptional regulators of adipogenesis such as PPARγ and C/EBPα. The expression of another protein, preadipocyte factor-1 (PREF-1), which is considered to be responsible for maintaining the phenotype of pre-adipocytes, decreases dramatically upon induction of adipocytes differentiation (Lazar et al., 2018).

Each year, approximately 10% of adipocytes turn over in human adipose tissue (Lowe et al., 2011). This long duration means that the proper functioning of these newly formed adipocytes must be ensured to prevent dysfunction and metabolic diseases (Lowe et al., 2011). Promotion of normal function of adipocytes, or replacement of poorly functioning adipocytes may prove beneficial in overcoming the problem of obesity and its associated disorders.

## Biology of White, Brown and Beige Adipose Tissues

**FIGURE 1:**
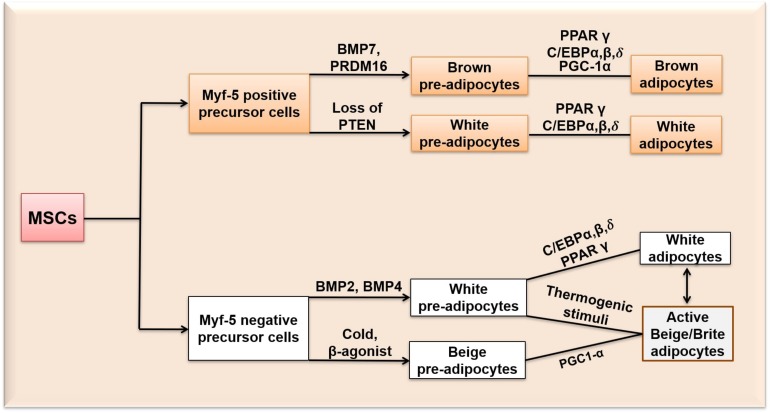
Differentiation of MSCs into white, beige/brite and brown adipocytes. Myf-5, Myogenic Factor-5 protein; BMP 7, Bone morphogenetic protein 7; BMP 2, Bone morphogenetic protein 2; BMP 4, Bone morphogenetic protein 4; PRDM 16, PR-domain containing 16; PGC-1α, peroxisome proliferator-activated receptor gamma coactivator 1-alpha; PPARγ, peroxisome proliferator-activated receptor gamma; PTEN, phosphatase and tensin homologue; C/EBPα,β,δ, CCCAAT/Enhancer Binding Protein α, β, δ.

There are two main types of adipose tissues in mammals; white and brown adipose tissues (WAT and BAT), characterized by different morphologies, anatomical locations, biochemical features, functions and gene expression patterns. Both are involved in the homeostasis of energy (Park, 2014). The main constituent of adipose tissue is WAT, which is used as an energy substrate when needed. WAT adipocytes have a greater average diameter (20–150 μm) than those of BAT (10–25 μm) (Stock and Cinti, 2003). White adipocytes contain a single lipid droplet of triglycerides (formed from esterification of fatty acids and glycerol-3-phosphate). WAT represents more than 95% of adipose mass while BAT represents 1–2% of the fat (Kahn et al., 2019). Brown adipocytes contain high numbers of multilocular lipid droplets as well as many mitochondria (Park, 2014). BAT is known to be protective against hypothermia due to its capacity to break down lipids to generate heat (thermogenesis). WAT stores triglycerides while BAT disperses energy in thermogenesis - thus there is a complementary functional relationship between the two forms (Coelho et al., 2013; Mukherjee et al., 2015). Mitochondria within BAT host key thermogenic protein uncoupling protein 1 (UCP1), which is a crucial player for thermogenesis (Tam et al., 2012; Shan et al., 2016). UCP1 is expressed in the inner membrane of mitochondria and is responsible for the generation of heat via respiratory uncoupling reactions. It converts chemical energy into heat via proton leak across the inner membrane of mitochondria (Park, 2014). The expression of UCP1 in WAT has also been reported previously: over-expression of the transcriptional activator (Zfp516) of UCP1 resulted in the ‘browning’ of WAT, giving what is known as beige or ‘brite’ (brown in white) adipocytes. Zfp516 is a novel transcriptional activator of UCP1 and can be induced by hormonal stimulation, exposure to cold, and innervation (Dempersmier et al., 2015). It directly binds to the proximal region of UCP1 promoter and interacts with transcriptional co-regulator PR-domain containing 16 (PRDM 16) to activate UCP1 promoter (Dempersmier et al., 2015). In addition to Zfp516, various other transcriptional regulators have also been implicated in the activation of brown/beige adipocytes specific genes (Shapira and Seale, 2019). These include interferon regulatory factor 4 (IRF4), Krüppel-like factor 11 (KLF11), TATA-binding protein associated factor 7L (TAF7L), zinc finger and BTB domain-containing protein 16 (ZBTB16), placenta-specific gene 8 protein (PLAC8), early B cell factor 2 (EBF2), forkhead box C2 (FoxC2) and ewing sarcoma break point region 1 (EWSR1) (Seale et al., 2009; Seale, 2015; Inagaki et al., 2016; Mueller, 2016; Shapira and Seale, 2019). Beige adipocytes are inducible and possess characteristics of both WAT and BAT (Herz and Kiefer, 2019). Under basal conditions, beige adipocytes in WAT show phenotypes similar to white adipocytes: they lack expression of UCP1 and contain one large lipid droplet (Petrovic et al., 2010). However, when exposed to cold (Barbatelli et al., 2010) and β3-adrenergic activators (Himms-Hagen et al., 2000), these beige cells acquire characteristics similar to brown adipocytes including expression of UCP1 and presence of small multilocular lipid droplets. Recruitment of beige adipocytes within WAT leads to the acquisition of thermogenic capacity in WAT, just like that of BAT (Chayama et al., 2019). Beige adipocytes can also be differentiated de novo from the dedicated white precursor cells, whenever stimuli such as β3-adrenergic activators or exposure to cold are met (Figure 1) (Harms and Seale, 2013; Rosen and Spiegelman, 2014; Merlin et al., 2016). However, they are converted back to white adipocytes when heat generation is no longer a priority (Rosen and Spiegelman, 2014), illustrating that these cells exhibit extraordinary plasticity in response to changes in the physiological conditions. The thermogenic activity of beige cells has been reported to act against obesity and increase energy expenditure (Crane et al., 2015). The prevalence of beige cells is in inverse proportion to obesity, body mass index, and plasma glucose level (Cypess et al., 2009), evidencing the importance of their role in the regulation of body’s metabolism. Some reports had stated that BAT was present only in newborns and small mammals, but recent studies have revealed conclusively the presence and functional relevance of BAT (Saely et al., 2011) and beige adipose tissue in adults (Shinoda et al., 2015).

Although both brown and white adipocytes originate from MSCs, it is believed that the immediate precursor cells giving rise to brown and white adipocytes are different (Figure 1): MSCs are pledged either to adipogenic (Myf-5 negative) cells giving rise to white adipocytes, or myogenic (Myf-5 positive) cells that become brown adipocytes (Timmons et al., 2007; Park, 2014). The Myf-negative precursor cells give rise to white pre-adipocytes through the expression of bone morphogenetic protein-2 and 4 (BMP 2 and 4) and beige pre-adipocytes upon exposure to cold or β3-adrenergic activators. BMP 2 is known to promote osteogenesis in human bone-marrow cells and white adipogenesis in mouse-derived 3T3-L1 and C3H10T1/2 cells by inducing the expression of PPARγ (Margoni et al., 2012). BMP 4 promotes the commitment of MSCs to the adipogenic lineage and is reported to induce adipogenesis in a dose-dependent manner in mouse embryonic stem cells (Taha et al., 2006). The Myf-positive precursor cells give rise to brown pre-adipocytes through bone morphogenetic protein-7 (BMP 7) expression and PRDM16 (Kajimura et al., 2009; Park, 2014). BMP 7 activates brown adipogenesis only through the p38MAPK pathway by inducing the expression of brown adipogenesis-specific transcriptional factors such as UCP1, Peroxisome proliferator-activated receptor gamma coactivator 1-alpha, beta (PGC-1α,β), C/EBPs and PPARγ (Tseng et al., 2008). PRDM16 is expressed both before and after adipocyte differentiation (Morganstein et al., 2010). Both PRDM16 and C/EBPβ together act as a switch in determining the fate of BAT away from myogenic lineage by inducing the expression of PGC-1α and PPARγ (Park, 2014). It has also been reported that myf expressing cells (myf-5 positive precursor cells) can be differentiated into white adipocytes (Harms and Seale, 2013; Rosen and Spiegelman, 2014). Sánchez-Gurmaches et al. (2012) evidenced a subset of white adipocytes which were derieved from myf-5 positive progenitor cells. Loss of phosphatase and tensin homologue (PTEN) in myf-5 positive precursors resulted in a subset of white adipocytes (Sánchez-Gurmaches et al., 2012). However, most of the evidence supports that BAT and WAT originates from different developmental paths (Rosen and Spiegelman, 2014). Recently, efforts have been made to identify the transcriptional mechanisms specific to WAT and BAT-related gene regulatory networks. It has been observed that most of the adipogenic factors, for example, PPARγ functions in the differentiation of both WAT and BAT but with binding sites specific to either WAT or BAT. For example, early beta-cell factor-2 and PRDM 16 recruits PPARγ to BAT selective genes, while TLE3 recruits PPARγ to activate specifically white adipogenesis (Giralt and Villarroya, 2013).

The recent rediscovery of effective BAT in adult humans has invigorated interest in it as a viable and novel target for anti-obesogenic drugs (Park, 2014). BAT transplantation studies have revealed that besides thermogenic activities, BAT also acts as an endocrine organ and secretes various brown adipokines known as batokines to orchestrate adaptive thermogenesis and, in turn, improving metabolic health (White et al., 2019). These batokines exert endocrine, autocrine and paracrine actions and target distant organs to exert their effects (Villarroya et al., 2019). Batokines include fibroblast growth factor-21 (FGF21), C-X-C motif chemokine ligand-14 (CXCL14), bone morphogenetic protein-8b (BMP8b), growth-and-differentiation factor-15 (GDF15), neuregulin-4 (NRG4), S100 calcium-binding protein b (S100b) and various others (Villarroya et al., 2019). These secreted batokines perform various functions and contribute to the regulation of immune activities, thermogenic activities, cardioprotective effects, vascularization, substrate utilization etc. (Lee et al., 2019; Villarroya et al., 2019). The activity of BAT in human is in inverse relation to the onset of obesity, type II diabetes and age (Lee et al., 2013). Upregulating the proteins and transcriptional factors specifically expressed in brown or beige adipocytes is a highly promising approach in the elimination of obesity. Activation of the thermogenic system in humans, either in WAT or BAT, should correlate well with an increase of energy expenditure. Thus, developing browning-inducing strategies in WAT or activation of BAT might contribute to a crucial strategy for treating obesity.

## Transcriptional Regulation of Adipogenesis

**FIGURE 2:**
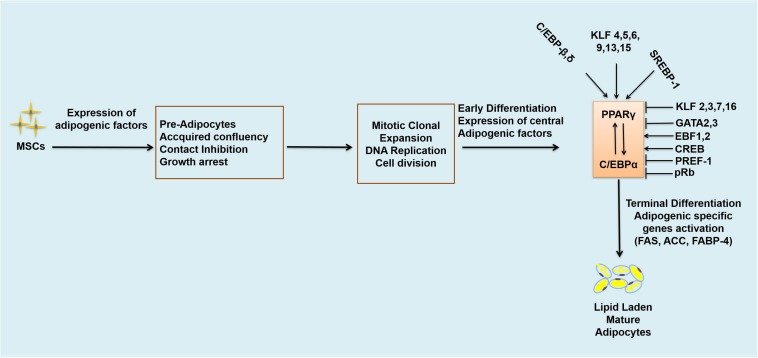
Transcriptional regulation of adipogenesis. Arrows represent activation and bars represent inhibition. MSCs, mesenchymal stem cells; DNA, deoxyribonucleic acids; C/EBPs, CCAAT/enhancer binding proteins. C/EBPβ,δ; CCCAAT/Enhancer Binding Protein β, δ, KLF 4,5,6,9,13,15, Kruppel-like factor 4,5,6,9,13,15; SREBP-1, Sterol regulatory binding protein-1; KLF 2,3,7,16, Kruppel-like factor 2,3,7,16; GATA 2,3, GATA binding protein 2,3; EBF-1, 2, Early β-cell factor 1,2; CREB, cyclic AMP response binding element; PREF-1, preadipocyte factor-1; pRb, retinoblastoma protein; FAS, fatty acid synthase; ACC, acetyl CoA carboxylase; FABP-4, fatty acid binding protein-4.

Adipogenesis is controlled by a large number of transcriptional factors, including C/EBP family members and PPARγ (Ali et al., 2013). Expression of C/EBPβ and C/EBPδ occurs at early stages of adipocyte differentiation and together they induce the expression of C/EBPα and PPARγ which are the central positive modulators of adipogenesis (Rosen et al., 2009; Khalilpourfarshbafi et al., 2018). C/EBPβ is considered the most important, being induced rapidly after the induction of adipogenic stimuli (Guo et al., 2015). Knockdown of C/EBPβ is reported to block adipogenesis in 3T3-L1 adipocytes (Zhang et al., 2011; Guo et al., 2012, 2015). PPARγ is the master regulator involved in the differentiation of adipocytes and metabolism (Lefterova et al., 2014). PPARγ and C/EBPα exert positive feedback on each other (Figure 2), co-operating to orchestrate the complete adipogenic program (Khalilpourfarshbafi et al., 2018). Several studies (Barak et al., 1999; Rosen et al., 1999) have indicated that PPARγ is the key regulator involved in the development and differentiation of adipocytes, and therefore known to be obligated for the differentiation of adipocytes; cells deficient in PPARγ cannot differentiate into mature adipocytes even if other powerful pro-adipogenic factors are ectopically expressed (Rosen et al., 2009). Previous *in vitro* studies have shown that most of the activators and repressors of adipogenesis alter the activity and expression of PPARγ (Sarjeant and Stephens, 2012). Transcriptional factors such as C/EBPβ, C/EBPδ, Kruppel-like factor 5 (KLF5) and early β-cell factor 1 (EBF1) are known to directly induce the expression of PPARγ mRNA in adipogenesis (Rosen et al., 2009). Early β-cell factor 1 and 2 (EBF1 and EBF2) are induced during the differentiation of the 3T3-L1 white pre-adipocyte cell line, but their pattern of expression is different from each other (Jimenez et al., 2007). EBF1 binds to the promoter of C/EBPα, directly activating C/EBPα and PPARγ (Jimenez et al., 2007). EBF2 has been reported to regulate brown adipocyte genes expression (Ucp1 and Prdm16) and is expressed at higher levels in BAT as compared to WAT (Rajakumari et al., 2013). Reduction of EBF1 and 2 blocks the differentiation of 3T3-L1 cells (Jimenez et al., 2007).

Likewise, other transcriptional factors also contribute to the regulation of adipogenesis. Kruppel-like factors (KLFs) may be either activators or suppressors of adipogenesis. KLF4, KLF5, KLF6, KLF9, KLF13 and KLF15 are known to enhance adipogenesis while KLF2, KLF3, KLF7 and KLF16 inhibit adipogenesis (Sue et al., 2008; Jiang et al., 2015; Jang et al., 2016; Pollak et al., 2018). Adenovirus-mediated ectopic expression of KLF2 has been reported to inhibit the expression of C/EBPα, PPARγ and sterol regulatory binding protein-1c (SREBP-1c) but did not have any effect on C/EBPδ and C/EBPβ expressions (Banerjee et al., 2003). KLF3 inhibits 3T3-L1 pre-adipocyte differentiation by repressing the C/EBPα promoter (Sue et al., 2008), as does KLF7 (Cho et al., 2007); overexpression of KLF7 significantly decreases C/EBPα, PPARγ, adipsin and aP2 expression (Kawamura et al., 2006). KLF16 overexpression inhibits the differentiation of 3T3-L1 cells and brown pre-adipocytes through downregulation of PPARγ expression, while knockdown of KLF16 promotes differentiation of both brown, and white adipocytes and increases expression of PPARγ (Jang et al., 2016). Similarly, GATA binding proteins 2 and 3 (GATA2 and 3) also decrease the rate of adipogenesis by downregulating PPARγ expression (Tong et al., 2005; Khalilpourfarshbafi et al., 2018). GATA2 and 3 are expressed predominantly in the pre-adipocytes of WAT, decreasing the expression of PPARγ2 – and hence adipogenesis - through direct suppression of PPARγ2 promoter, and formation of inhibitory complexes with C/EBP family members. GATA2 and 3 are also known to have a role as molecular gatekeepers during the differentiation of adipocytes and may be novel targets for preventative anti-obesogenic therapies (Feng et al., 2016). Another protein, PREF1, is expressed abundantly in pre-adipocytes, but its expression decreases significantly upon the development of adipocytes. Ectopic expression of PREF1 inhibits 3T3-L1 differentiation and reduces the expression of C/EBPα and PPARγ (Rosen and MacDougald, 2006; Sarjeant and Stephens, 2012). Mice deficient in PREF1 showed retarded growth and enhanced adiposity (Sarjeant and Stephens, 2012). Other transcriptional factors such as cyclic AMP response binding element (CREB) and sterol regulatory binding protein-1 (SREBP1), (which expedites metabolism of fatty acids by inducing expression of PPARγ) are positive regulators of adipogenesis and needed in the differentiation of white pre-adipocytes into mature adipocytes (Khalilpourfarshbafi et al., 2018). In white pre-adipocytes CREB is required for the induction of differentiation of adipocytes and absence of CREB inhibits pre-adipocytes differentiation (Reusch et al., 2000). CREB is required to induce the expression of C/EBPβ during the early stages of adipocyte differentiation. It binds to the dual *cis* regulatory elements (TGA1 and TGA2) within the proximal promoter region of C/EBPβ gene and activates its transcription. Expression of a dominant-negative CREB in mouse embryonic fibroblasts (MEFs) has been observed to block adipogenesis and expression of C/EBPβ (Zhang et al., 2004). SREBP1 is also involved in adipocytes differentiation, may induce the expression of PPARγ and metabolism of fatty acids (Khalilpourfarshbafi et al., 2018). SREBP-1c is the highly expressed form of SREB1 in adipocytes (Sewter et al., 2002; Payne et al., 2010) and involved in the regulation of genes responsible for the synthesis of fatty acids such as FAS (Rosen et al., 2009). In addition, retinoblastoma protein (pRb) is also known to positively regulate white adipogenesis and is the founding member of the pocket proteins family (Hansen et al., 2004). It is considered as an essential player in the differentiation of white adipocytes in mice (Moreno-Navarrete et al., 2013). Activation of pRb positively regulates terminal differentiation of white adipocytes and inhibits brown adipogenesis (Chen et al., 1996; Moreno-Navarrete et al., 2013; Petrov et al., 2015). The adipogenic effects of pRB are due to its regulatory effects on memebers of C/EBPs family, especially C/EBPβ. It binds and augments the activity of C/EBPβ and hence positively regulates white adipogenesis. pRb-deficient fibroblasts are unable to undergo adipose conversion (Hallenborg et al., 2009). pRb also regulates fate choice and lineage commitment (Calo et al., 2010). Lack of pRb switches the cell fate from white to brown adipocyte, increases energy expenditure and acts as molecular switch in the determination of white versus brown adipogenesis (Hansen et al., 2004; Dali-Youcef et al., 2007). Inactivation of pRb in mouse embryonic fibroblasts, white pre-adipocytes and mouse stem cells resulted in increase brown adipogenesis and increased expression of UCP1 (Hansen et al., 2004) which demonstrates that activation of pRb is positively associated with white adipogenesis and its ablation promotes brown/beige adipogenesis.

As, both PPARγ and C/EBP family members are the central modulators of adipogenesis and are widely studied targets in *in vitro* and *in vivo* studies of anti-obesogenic medicine, so, insights into various signaling pathways, energy sensing proteins (e.g., AMPK), genes and their transcriptional factors which have direct interactions with PPARγ and C/EBP family members are required to tackle abnormal adipose tissue development and obesity pandemic.

## Role of Signaling Pathways in Adipogenesis

MSCs are committed to either osteogenic, myogenic or adipogenic lineages. This involves discrete signaling pathways including those of Bone morphogenetic protein (BMP), Wnt (canonical and non-canonical), and Hedgehog. These pathways exert very strong influences on the central regulators of both osteogenesis (Runx2) and adipogenesis (PPARγ), working antagonistically with overexpression of one factor repressing the other (Zhang et al., 2006; James, 2013).

### Transforming Growth Factors-β Pathway and Bone Morphogenetic Proteins

**FIGURE 3:**
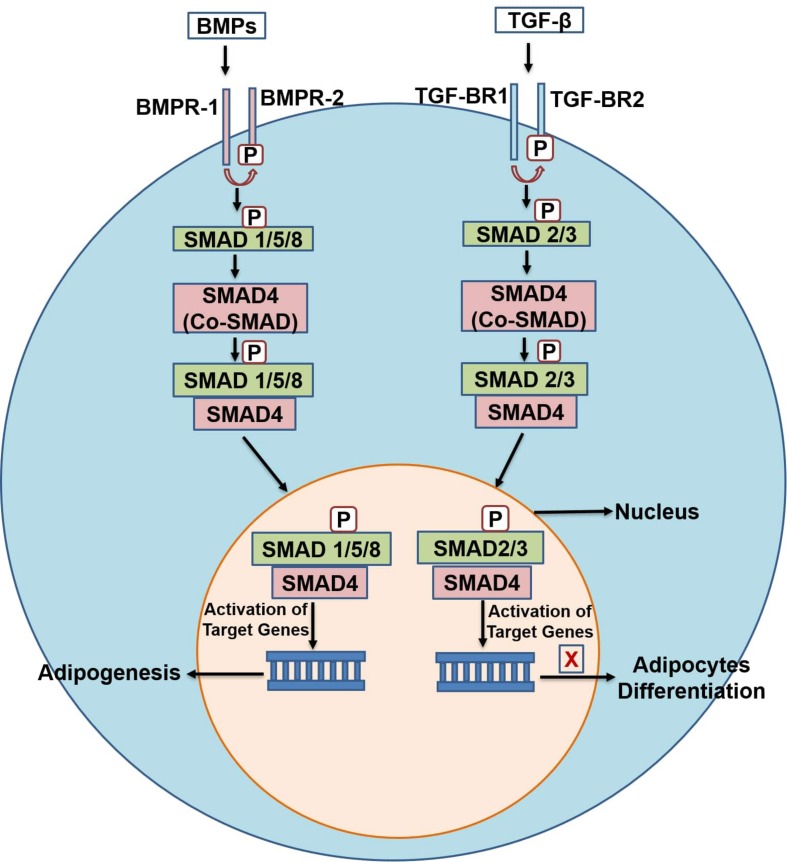
A schematic diagram of the TGF-β and BMPs pathway. TGF-β, transforming growth factor-beta; TGF-β-R1,2, transforming growth factor-beta receptor type 1, 2; co-SMAD, common-SMAD; BMPs, bone morphogenetic proteins; BMP-R1,2, bone morphogenetic protein- type 1 and 2 receptors.

The transforming growth factors-β (TGF-β) pathway consists of more than 33 members. These include TGF-β1, 2, and 3, bone morphogenetic proteins (BMPs), activins, nodal-related proteins and growth differentiation factors (GDFs) (Lee, 2018). Members of the TGF-β super family control diverse process such as cell differentiation, growth and cell fate specification (Budi et al., 2017; Lee, 2018) in various cell types including adipocytes (Margoni et al., 2012). The TGF-β pathway stimulates proliferation of pre-adipocytes but inhibits differentiation of pre-adipocytes into mature adipocytes (Zamani and Brown, 2010; Lee, 2018). Among all the TGF-β superfamily members, TGF-β1 has the greatest role in adipogenesis, inhibiting 3T3-L1 pre-adipocyte differentiation (Margoni et al., 2012) by interacting and repressing PPARγ, C/EBPα and C/EBPβ (Moseti et al., 2016). The signal transduction in TGF-β pathway begins when the TGF- β ligands bind to type 1 and 2 receptors (TGF-β-R1 and TGF-β-R2) present on the cell surface. These receptors are serine/threonine kinases and convey the signals through downstream processes (Margoni et al., 2012). TGF-β ligands bind to the TGF-β-R2 receptor and recruit (phosphorylate) the TGF-β-R1 receptor. The phosphorylated TGF-β-R1 receptor then targets and potentiates the downstream specific receptor-regulated SMAD proteins referred as R-SMADs. In TGF-β branch of the pathway two R-SMADs (SMAD2/3) take part in the process. These R-SMADs upon phosphorylation associate with common-SMAD (co-SMAD/SMAD4) and form a heterocomplex. This complex then translocates to the nucleus and activates the target genes (Lee, 2018) (Figure 3).

Bone morphogenetic proteins (BMPs) also belong to the TGF-β superfamily and have been identified as regulators of osteogenesis, and more recently, adipogenesis (James, 2013; Moseti et al., 2016), as well as having regulatory roles in proliferation, apoptosis, differentiation, and determination of cell fate in adulthood and during embryogenesis (Chen et al., 2004; Wang et al., 2014; Moseti et al., 2016). In contrast to the TGF-β pathway, BMPs are generally considered as stimulators of both white and brown adipogenesis. Their signal transduction is similar to the TGF-β branch, with differences in the cell surface receptors and types of SMAD proteins involved. Signal transduction begins when BMPs ligands bind to cell surface receptors BMPR-1 and BMPR-2. These liganded receptors then activate (phosphorylate) the R-SMADs (SMAD 1/5/8), which associates with co-SMAD (SMAD4), translocate to the nucleus and activate target genes (Figure 3). BMPs play different roles in the differentiation of adipocytes depending on the stage of cells, BMP type and dosage (Tseng and He, 2007). BMP-2 and 4 have been shown to commit pluripotent stem cells toward the adipogenic lineage (Tang and Lane, 2012). BMP-7 has been shown to activate differentiation toward brown adipocytes (Tseng et al., 2008). 3T3-F44 2A pre-adipocytes treated with BMP-2 showed a decrease in insulin-induced lipid accumulation (Skillington et al., 2002), but BMP-7 increased the differentiation of 3T3-L1 pre-adipocytes, demonstrating the contradictory roles of BMPs in adipogenesis (Rebbapragada et al., 2003; Suenaga et al., 2013). Similarly, BMP-4 regulates the commitment of precursor cells into white adipogenic lineage (Gustafson and Smith, 2012). BMP-4 and BMP-7 can also activate the development of beige adipocytes in human precursor cells (Elsen et al., 2019). Overexpression of BMP-4 in transgenic mice showed a reduction in the size and mass of WAT and induced browning of WAT (Yu et al., 2013). Induced expression of BMP-4 upregulated the expression of key regulators of brown adipose tissue, peroxisome proliferator-activated receptor gamma coactivator 1-α (PGC-1α) and its target gene, UCP1 (Qiang et al., 2007; Smith and Kahn, 2016). Likewise, BMP-8b and BMP-9 are also known to promote brown adipogenesis. BMP-8b is known to enhance energy dissipation in the body (Pellegrinelli et al., 2018). It regulates energy metabolism by increasing BAT thermogenesis both centrally through activation of AMPK and peripherally through activation of p38 MAPK pathway in mature and differentiating brown adipocytes (Whittle et al., 2012; Pradhan et al., 2017). BMP-8b over expression increases the browning of subcutaneous WAT and enhances its thermogenic capacity (Pellegrinelli et al., 2018). Whittle et al. (2012) reported that mice with BMP-8b deletion (BMP-8b^–/–^) exhibited impaired thermogenesis and reduced metabolic rate. BMP-9 has been reported to enhance brown adipogenesis in human adipose-derived stem cells (hASCs). Kuo et al. (2014) showed that a recombinant BMP-9 derivative (MB109) induced the thermogenic UCP1 gene mRNA expression and enhanced brown adipogenesis in hASCs, thus shows anti-obesogenic capacity. These pathways (TGF-β and BMP) are therefore of great interest for the discovery of novel chemotherapies in preventing obesity through inhibition of WAT development, and promotion of BAT by targeting the key regulating factors of these pathways.

### Wnt Signaling Pathways

**FIGURE 4:**
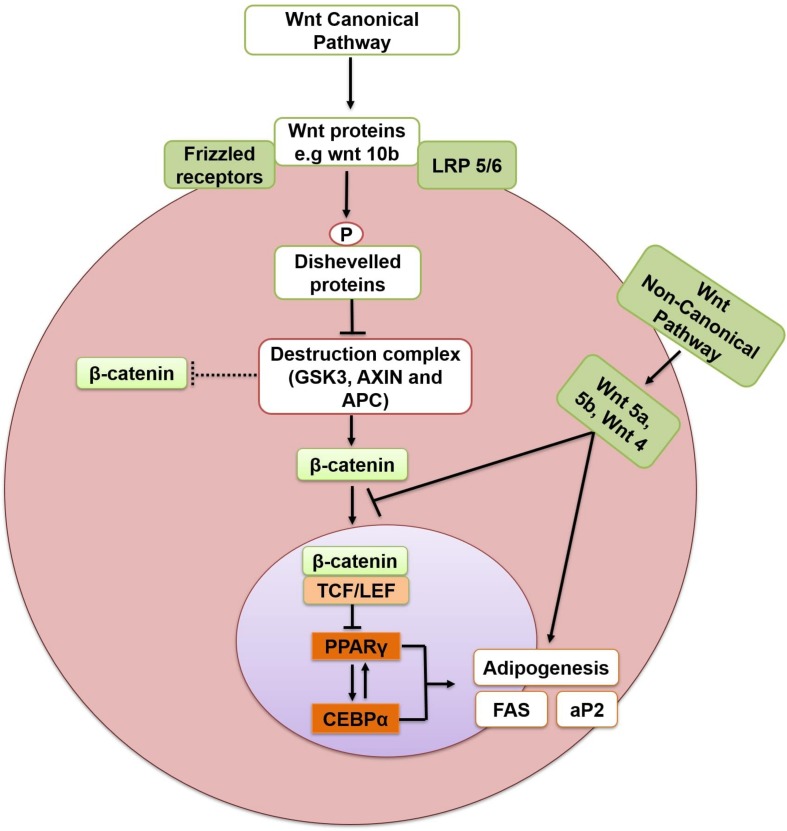
Inhibition of adipogenesis through Wnt/β catenin dependent pathway. Arrows indicate activation and bars indicate inhibition. LRP 5/6, lipoprotein receptor-related protein 5/6; GSK-3, glycogen synthase kinase-3; APC, adenomatous polyposis coli;TCF/LEF, T-cell factors/lymphoid-enhancing factor; PPARγ, peroxisome proliferator-activated receptor gamma; C/EBPα, CCCAAT/Enhancer Binding Protein alpha; FAS, fatty acid synthase; aP2, adipocyte fatty acid binding protein.

Wnts (Wingless-type MMTV integration site family members) are secreted glycoproteins that work both in an autocrine and paracrine manner (Moseti et al., 2016) and are post-translationally modified by the addition of lipids (Hu et al., 2018). Wnt signaling refers to a group of conserved signal transduction pathways consisting of proteins which convey signals through cell surface receptors into the cell. These pathways are involved in cell differentiation and proliferation in adult tissue regeneration, and in embryonic development (Moseti et al., 2016). Abnormal activities of various appendages in Wnt signaling pathways causes the aberrant expansion of adipose tissue (Aamir et al., 2019). Wnt pathways can be divided into canonical (β-catenin dependent) and non-canonical (β-catenin independent) pathway. MSCs are differentiated into osteocytes and myocytes instead of adipocytes upon Wnt/β-catenin signaling pathway activation. Conversely, interruption of Wnt/β-catenin signaling promotes adipogenesis (Figure 4) (Li et al., 2007). Wnt/β-catenin plays a negative regulatory role in confining the differentiation of adipocytes (Aamir et al., 2019). The signal transduction begins when Wnt proteins (e.g., wnt 10b) attach to the Frizzled receptors and lipoprotein receptor-related protein 5/6 (LRP5/6) to form a heterotrimeric complex. This complex phosphorylates (activates) Disheveled proteins which disrupt the destruction complex containing Glycogen synthase kinase-3 (GSK-3)-AXIN- adenomatous polyposis coli (APC) (GSK-3-AXIN-APC), which would otherwise degrade β-catenin. Inhibition of the destruction complex releases and stabilizes β-catenin in the cytoplasm. β-catenin then translocates to the nucleus, attaches to T-cell factors/lymphoid-enhancing factor (TCF/LEF) and inhibits adipogenesis through suppression of PPARγ and C/EBPα. Wnt10b is one of the important element of the Wnt/β-catenin pathway. It has been reported to be responsible for the anti-adipogenic function of canonical pathway. Wnt10b is highly expressed in pre-adipocytes, but its expression declines promptly after induction of differentiation (Bennett et al., 2002). Its overexpression stabilizes cytoplasmic β-catenin and blocks adipogenesis in 3T3-L1 pre-adipocytes (Krishnan et al., 2006; Christodoulides et al., 2009). Similarly, the other two members of the canonical pathway, Wnt6 and Wnt10a have also been shown to promote osteogenesis and inhibit adipogenesis in St2 and 3T3-L1 pre-adipocytes (Cawthorn et al., 2012). Moreover, the Wnt/β-catenin signaling pathway also inhibits brown adipogenesis by disrupting the PPARγ and C/EBPα induction. Wnt10a and Wnt10b, members of the canonical pathway, are the possible endogenous inhibitors of BAT. Both Wnt10b and Wnt10a are expressed in pre-adipocytes of BAT but not in differentiated brown adipocytes and their expression reduces with the progression of brown adipogenesis (Kang et al., 2005). In addition, Wnt signaling also blocks the thermogenic program of BAT by suppressing the thermogenic protein, UCP1 of BAT through repression of PGC-1α (Kang et al., 2005). *In vivo* expression of Wnt10b from fatty acid-binding protein 4 (FABP 4) promoter had been shown to reduce total body fat by 50% and provide resistance to WAT accumulation in high-fat diets (Longo et al., 2004).

Members of Wnt family also activate the non-canonical β-catenin independent pathway. As compared to canonical pathway, less is known about non-canonical β-catenin independent pathway. Members of this pathway include Wnt 4, 5a/b, 6, 7a/b, and 11 (Ackers and Malgor, 2018). Activation of the non-canonical pathway through Wnt5a is reported to antagonize the canonical pathway, promoting the differentiation of pre-adipocytes (Topol et al., 2003). Similarly, Wnt4 and Wnt5a promote differentiation of adipocytes (Nishizuka et al., 2008), and Wnt5b together with Wnt5a is shown to inhibit the Wnt/β-catenin signaling and promotes adipogenesis by activating PPARγ (Van Tienen et al., 2009). Accordingly, adenoviral overexpression of the related Wnt5b impaired β-catenin nuclear translocation and enhanced 3T3-L1 cell differentiation (Kanazawa et al., 2005). The non-canonical pathway, therefore, antagonizes the canonical pathway and promotes adipogenesis.

Since the Wnt/β-catenin dependent pathway inhibits adipogenesis and directs the cells toward osteogenesis or myogenesis, its activation constitutes an attractive drug-development target to combat obesity and the associated metabolic complications.

### Hedgehog Signaling Pathway

**FIGURE 5:**
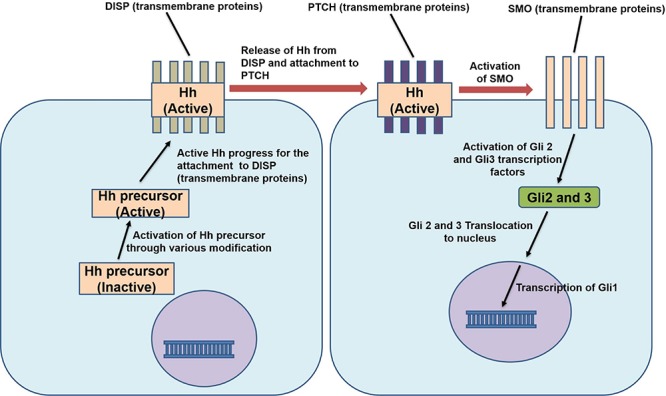
Mechanism of action of Hedgehog signaling pathway. Hh, Hedgehog protein; DISP, dispatched protein; PTCH, patched protein; SMO, Smoothened protein; Gli 1,2,3, glioblastoma gene 1,2,3.

The Hedgehog (Hh) signaling pathway was first discovered in Drosophila but is now known to be involved in the development of all vertebrates (Liang et al., 2015). The proteins of the Hh family are known as Sonic Hedgehog (SHH), Desert Hedgehog, and Indian Hedgehog (IHH) and participate in the same highly conserved Hh signaling pathway (James, 2013) which is an important modulator of stem cell differentiation. Notably, its role in the differentiation of MSCs has been demonstrated in several studies (Fontaine et al., 2008; Plaisant et al., 2009). Hh signaling initiates when the insoluble and inactive Hh polypeptide precursor is converted to a soluble (active) form which makes it capable of diffusing across the cell membrane. This modified protein then secreted from cell transmembrane proteins named Dispatched (DISP). After secretion, the Hh polypeptide binds to another cell surface receptor Patched (PTCH) present on nearby cells. This binding releases another protein called Smoothened (SMO), suppressing the PTCH, thus enabling them to activate the glioblastoma gene product (Gli1-3) (James, 2013). Glis are the core transcription factors of this pathway (Figure 5); Gli1 is used as a reliable marker for the activity of Hh signaling (Tzameli et al., 2004). Hh signaling has inhibitory effects on adipogenesis in murine cells, i.e., KS483, mouse adipose-derived stromal cells, C3H10T1/2 and calvaria MSC lines (Tang and Lane, 2012). In mammalian fat, the levels of the components of Hh signaling respond dynamically to adipogenesis and obesity (Suh et al., 2006). In mice, subcutaneous fat pad and WAT decrease when the Hh pathway is activated (Li et al., 2008). Fan et al. (2018) reported that Hh signaling primarily acts on the later stages of adipocytes differentiation in porcine adipose-derived MSCs. It was revealed that the expression pattern of Gli1, C/EBPα and PPARγ were changed on the fourth day of activation of the pathway. Gli1 mRNA and protein expression reached the maximum on the fourth day before gradually decreasing. The mRNA and protein expression of C/EBPα and PPARγ were suppressed significantly on the fourth day of activation of Hh signaling pathway. Reduced expression of Gli1, 2, 3 and PTCH promote adipogenesis in MSCs (Fontaine et al., 2008; James, 2013). This signaling pathway is downregulated during the differentiation of human adipocytes and upon activation, it reduces the expression of C/EBPα and thus hinders the accumulation of lipids and adipogenesis (Fontaine et al., 2008; Moseti et al., 2016). Hh signaling pathway activation in C3H10T1/2 mouse cell lines was reported to inhibit PPARγ and C/EBPα expression, blocked the differentiation of pre-adipocytes and increased the commitment of C3H10T1/2 mouse cell lines toward osteogenic lineage (Spinella-Jaegle et al., 2001). Activation of Hh gene in a *B. mori* cell line (BmN) inhibited aP2 expression, while knockdown of the Hh gene by RNA interference enhanced aP2 gene expression indicating the regulatory effect of Hh on aP2. Moreover, the blocking of the Hh signaling pathway by an antagonist, cyclopamine, in silkworm larvae resulted in increased differentiation and size of adipocytes. Inhibition of fat formation by Hh signaling pathway was retained both in vertebrates and invertebrates (Liang et al., 2015). While it has been revealed that activation of Hh signaling impairs adipogenesis, it is also counterintuitively reported that decrease or blockade of Hh pathway is necessary but not sufficient to trigger adipocyte differentiation (Fontaine et al., 2008; Fan et al., 2018).

## AMPK and its Activation in Adipose Tissue

AMPK is a serine/threonine kinase which is expressed in different kinds of tissues (liver, adipose, skeletal, kidney and hypothalamus) (Kim and Park, 2016) and plays a vital role in controlling and regulating cell cycle and cellular energy homeostasis. AMPK is a fuel-sensing enzyme – it is involved in sensitivity to, and the homeostasis of, lipids, glucose and insulin (Xu et al., 2012). AMPK activation results in an increase in the body’s cellular energy levels (Kim and Park, 2016). This heterotrimeric protein consists of 3 subunits: catalytic subunit α which is comprised of two further subunits α1, α2 and regulatory subunits β and γ consisting of two subunits (β1, β2) and three subunits (γ1, γ2, γ3) respectively (Kim et al., 2016; Hardie, 2018). In adipose tissue, the α1 subunit is considered to be the most important subunit and accounts for the majority of the activity of AMPK (Daval et al., 2006). Stimuli such as exercise, fasting, undernutrition, and exposure to cold result in activation of AMPK in adipose tissue (Daval et al., 2005). For example, in C557Bl/6 mice, AMPK activation increased in BAT in response to chronic (>7 days) cold exposure, and in WAT the activity of α1 AMPK was increased by almost 98% after exposure to cold for more than 15 days (Mulligan et al., 2007). Endogenous stimulators such as high-density lipoproteins (HDLs), β-adrenergic stimulators, eicosapentaenoic acid and homocysteine also activate AMPK in BAT of rats and mice, and 3T3-L1 adipocytes (Bijland et al., 2013) as does IL 6 in adipose tissue (Daval et al., 2006). Decreased phosphorylation of AMPK was found in adipose tissue of IL 6 knockout mice after heavy exercise (Daval et al., 2006). The high concentration of adenosine monophosphate (AMP) and low levels of adenosine triphosphate (ATP) resulting from stimuli such as nutrient deprivation, ischemia and hypoxia activate AMPK allosterically through regulation of an upstream kinase of AMPK (Daval et al., 2006; Katwan et al., 2019). In the case of low ATP and high AMP levels, the upstream kinase of AMPK; liver kinase b1 (LKB1) is activated and phosphorylates AMPK (Bijland et al., 2013). Similarly, the adipokines adiponectin and leptin also activate AMPK in adipose tissue (Daval et al., 2006). Overexpression of adipose-specific leptin receptor in WAT of mice leads to an increase in phosphorylation of AMPK Thr^172^, showing that leptin also activates AMPK in adipose tissue (Wang et al., 2005). Orci et al. (2004) reported the phosphorylation of AMPK in hyperleptinemia white adipocytes. These adipocytes were transformed into “fat burning machines” and it appeared that the combustion of fat was due to the leptin-induced phosphorylation (activation) of AMPK along with increased expression PGC-1α and other thermogenic proteins and reduced expression of lipogenic proteins. Similarly, activation of AMPK by adiponectin in epididymal rat adipocytes is reported by Wu et al. (2003). Cellular treatment with adiponectin increased the phosphorylation of AMPK at Thr^172^ and its downstream target ACC and resulted in increased glucose uptake. Inhibition of AMPK activation by pharmacological agents abrogated glucose uptake indicating the activation of AMPK by adiponectin.

### Metabolic Functions of AMPK and Role in Adipogenesis

**FIGURE 6:**
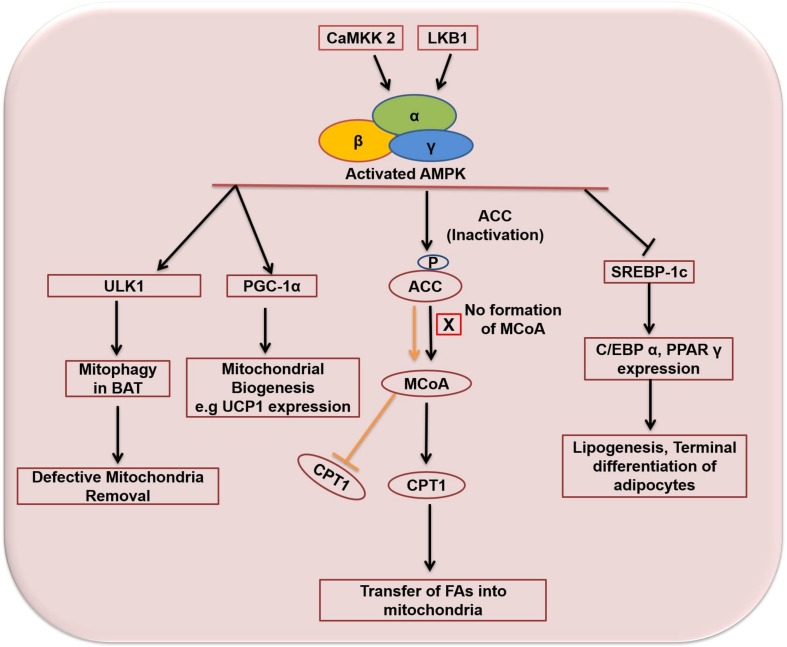
Activation and functions of AMPK in adipose tissue. Orange arrow and bar indicates functions of ACC and MCoA in the absence of AMPK activation. Arrows indicate activation and bars indicate inhibition. ACC, acetyl-CoA carboxylase; MCoA, malonyl co-enzyme A; CPT1, carnitine palmitoyltransferase 1; CaMKK2, calcium/calmodulin-dependent protein kinase kinase 2; LKB1, liver kinase b1; ULK1, Unc-51 Like Autophagy Activating Kinase 1; UCP1, uncoupling protein 1; PGC-1α, peroxisome proliferator-activated receptor gamma coactivator 1-alpha; SREBP-1c, sterol regulatory element binding protein 1; C/EBPα, CCCAAT/Enhancer Binding Protein alpha; PPARγ, Peroxisome proliferator-activated receptor gamma.

AMPK regulates lipid/glucose homeostasis, mitochondrial biogenesis, autophagy, protein homeostasis, redox equilibrium, food intake and insulin signaling (Ceddia, 2013; Jeon, 2016). Once activated, AMPK directly or indirectly promotes the phosphorylation of downstream targets, including transcription and translational factors, metabolic enzymes, epigenetic factors, growth and proliferation pathways. The overall effect of this regulation is to reduce the synthesis of cholesterol, fatty acids, ribosomal RNAs (rRNAs) and proteins (Yan et al., 2018). Regulation of lipid metabolism is the first known function of AMPK – its activation correlates with decreased lipid storage (Bijland et al., 2013). AMPK inhibits *de novo* synthesis of cholesterol, triglycerides (TG), and fatty acids (FAs), and activates FA uptake and β-oxidation (FAO). It inhibits and phosphorylates targets involved in the synthesis of fatty acids such as FAS, ACC1, and SREBP-1c (Figure 6). SREBP-1c is involved in the transcriptional regulation of various lipogenic enzymes, including FAS and ACC1. ACC1 is the predominant form of ACC expressed in lipogenic tissues (Oh et al., 2005; Ridgway and McLeod, 2015). ACC1 converts acetyl-CoA to malonyl-CoA and catalyzes the rate-limiting step in the synthesis of FAs (Luo et al., 2011; Jeon, 2016). Malonyl-CoA inhibits carnitine palmitoyl transferase 1 (CPT1) which is the rate-limiting enzyme for the transport of fatty acids to mitochondria for subsequent oxidation (Bijland et al., 2013). AMPK inhibits the synthesis of cholesterol by phosphorylating and inhibiting HMG-CoA reductase (Jeon, 2016). AMPK also stimulates mitochondrial biogenesis and β-oxidation through regulation of PGC-1α activity (Seo et al., 2015). Expression of PGC-1α is related to mitochondrial biogenesis whereas loss of PGC-1α function results in reduced expression of mitochondrial and thermogenic genes in WAT (Kleiner et al., 2012). Wan et al. (2014) reported that the induction of PGC-1α and the expression of mitochondrial proteins is regulated by AMPK in mouse epididymal adipose tissue. AMPK also regulates carbohydrate metabolism in liver, skeletal muscle and adipose tissue (Kola et al., 2008; Ceddia, 2013; Jeon, 2016). Skeletal muscle is the principal site of insulin-mediated glucose uptake (Koistinen and Zierath, 2002). In skeletal muscle, AMPK increases glucose uptake through increased glucose transporter type-4 (GLUT-4) translocation (Daval et al., 2006; Jeon, 2016). Exercise-stimulated glucose uptake in skeletal muscle is known to be mediated through the activation of AMPK (Kola et al., 2008). In addition, AMPK also attenuates glycogen synthesis through inhibition of glycogen synthase (GS) and activates glycogenolysis through activation of glycogen phosphorylase (GP) (Jeon, 2016). In adipose tissue the potential role of AMPK activation on glucose uptake is less clear (Bijland et al., 2013). The majority of the studies demonstrate that activation of AMPK in white adipocytes inhibits insulin-stimulated glucose uptake (van Dam et al., 2015a). However, some studies have reported an activating effect of AMPK on glucose uptake in adipose tissue. Ye et al. (2006) reported enhanced glucose uptake through activation of AMPK by rosiglitazone in adipose tissue and muscles. Similarly, Attané et al. (2011) and Shen et al. (2014) reported the effect of AMPK activation on glucose uptake in human adipose tissue and 3T3-L1 adipocytes. Activation of AMPK by apelin in human adipose tissue (Attané et al., 2011) and by cinnamon extract in 3T3-L1 adipocytes (Shen et al., 2014) enhanced glucose uptake. Inhibition of AMPK by compound-C showed opposite effect which indicates regulation of glucose uptake by AMPK in human adipose tissue and 3T3-L1 adipocytes.

In adipose tissue, indirect evidence suggests that activation of AMPK inhibits differentiation of white pre-adipocytes (Daval et al., 2006). AMPK regulates aP2 and induction of C/EBPs and PPARγ. AMPK has been shown to inhibit adipogenesis via inhibition of the early mitotic clonal expansion (MCE) phase accompanied by reduced expression of early and late adipogenic factors including FAS, SREBP-1c and aP2 (Habinowski and Witters, 2001; Bijland et al., 2013). Vingtdeux et al. (2011) reported the inhibition of adipogenesis by small-molecule activators (RSVA314 and RSVA405) of AMPK via MCE phase inhibition accompanied by reduced C/EBPβ expression, inhibition of C/EBPα, PPARγ and late adipogenic factors including SREBP-1c, FAS and aP2. Similarly in another study, AMPK activation by A769662 resulted in the reduction of lipid droplets and activation of PPARγ, C/EBPα, and early adipogenic transcription factors such as C/EBPβ and C/EBPδ (Zhou et al., 2009). Likewise, Moreno-Navarrete et al. (2011) showed reduced expression of key adipogenic factors such as FASN, ACC, PPARγ through activation of AMPK by metformin in human white preadipocytes differentiation. Moreover, it was observed that increased action of metformin was due to the increased expression of organic cation transporter 1 (OCT1 gene). Cotreatment with cimetidine, an OCT1 gene blocker, reversed the process resulting in increased adipogenesis and blunted AMPK activity. In addition, He et al. (2013) also reported the inhibition of adipogenesis through activation of AMPK. AMPK activation attenuated the expression of C/EBPα,β and PPARγ accompanied by decreased expression of SREBP-1c. The phosphorylation of ACC1 and expression of the rate-limiting enzyme CPT1 was also increased. These effects were reversed by using AMPK siRNAs, confirming the inhibitory function of activated AMPK in 3T3-L1 adipocytes. Similarly, Ono and Fujimori (2011) also showed the inhibition of adipogenesis through AMPK activation in 3T3-L1 pre-adipocytes. Pollard et al. (2019) reported that activation of AMPK protects against diet-induced obesity through thermogenesis. Chronic genetic activation of AMPK resulted in increase of whole-body energy expenditure which could be due to an increase in the consumption rate of oxygen in WAT. AMPK also regulates autophagy (Lee et al., 2018). Several studies have demonstrated autophagy in lipophagy, glycophagy, adipose tissue differentiation and mass regulation (Singh et al., 2009). AMPK regulates autophagy by phosphorylating two initiating regulators of autophagy: a protein kinase complex ULK1 and lipid kinase complex PI3KC3/VPS34 (Kim et al., 2013).

AMPK is also known to have an anti-inflammatory role in adipocytes (Mancini et al., 2017) and plays a key role in the inhibition of inflammatory responses (Morita et al., 2018). Inflammation in adipose tissue is known to cause obesity-induced insulin resistance (Makki et al., 2013). In obesogenic conditions, the hypertrophied adipocytes and the adipose tissue-resident immune cells increase the levels of circulating proinflammatory cytokines. Activation of AMPK in adipocytes rapidly suppresses the pro-inflammatory pathways (Mancini et al., 2017). Cheng et al. (2019) reported that catechin attenuates TNF-α stimulated inflammation through activation of AMPK/SIRT1 pathway in 3T3-L1 adipocytes. Similarly, Morita et al. (2018) showed that activation of AMPK reduced the release of MCP-1, which is known to be one of the most important pro-inflammatory adipocytokines. Its over expression in adipose tissue contributes to infiltration of macrophages and causes chronic low grade inflammation in adipose tissue (Kamei et al., 2006; Kanda et al., 2006; Morita et al., 2018). Mancini et al. (2017) also showed the anti-inflammatory effects of AMPK in 3T3-L1 adipocytes. Activation of AMPK inhibited the interleukin 1-β (IL 1-β) stimulated C-X-C motif chemokine 10 (CXCL10) secretion. CXCL10 is a proinflammatory cytokine and its upregulation correlates positively with obesity and type-2 diabetes (Zhang et al., 2014). Activation of AMPK also inhibited the TNF-α stimulated IKK/IκB/NFκB signaling (Mancini et al., 2017) which indicates the anti-inflammatory role of AMPK in adipocytes.

AMPK is obligatory for the proper functioning of BAT as well (Day et al., 2017). Activation of AMPK increases during the differentiation of brown adipocytes, and targeting AMPK with short interfering RNAs (siRNAs) inhibits the differentiation of pre-adipocytes into mature brown adipocytes (Vila-Bedmar et al., 2010; Bijland et al., 2013). AMPK is activated in BAT in a situation of chronic cold exposure, providing a thermogenic response (van Dam et al., 2015a). AMPK is integral to the browning of WAT, increasing energy expenditure through thermogenesis (Chung et al., 2017; Desjardins and Steinberg, 2018). It is vital for maintaining the mitochondrial structure, functions, and markers of mitophagy in BAT. Deletion of AMPK in mice brown and beige adipose tissue causes intolerance to cold exposure and reduces thermogenesis in response to β-adrenergic stimulation (Mottillo et al., 2016). These defects were due to impaired mitophagy which resulted in defective BAT mitochondria, non-alcoholic fatty liver disease (NAFLD) and insulin resistance. AMPK causes mitophagy through phosphorylation of Unc-51 like autophagy activating kinase 1 (ULK1) (Sinha et al., 2015; Mottillo et al., 2016).

### AMPK Activation by Upstream Kinases in Adipocytes

Under different physiological conditions, the subunits of AMPK behave differently and are regulated differently. Activation of AMPK can be achieved by either through upstream kinases or allosterically through AMP (Kim and Park, 2016). The best-studied mechanisms of the activation of AMPK are allosteric activation by binding of either AMP or ADP at γ subunit and by phosphorylation of the α subunit (Hardie et al., 2012). Conditions including hypoxia, exercise, ischemia and hypoglycaemia usually alter the cellular adenine nucleotides levels (suppress ATP consumption) and subsequently enhance the activity of AMPK (Hardie et al., 2003). The rise in AMP/ADP and decline in the levels of ATP cause the activation of AMPK by direct binding of ADP or AMP to the γ subunit of AMPK. This binding prevents access of phosphatases to Thr^172^ in the α subunit, and thus maintains a high phosphorylation level of AMPK (Jeon, 2016).

Upstream kinases of AMPK include LKB1, mouse protein 25 (MO25) and STE-related adaptor (STRAD), calcium/calmodulin-dependent protein kinase kinase (CaMKK) and transforming growth factor-β-activated protein kinase 1 (TAK1) (Oakhill et al., 2012; Desjardins and Steinberg, 2018; Wang et al., 2018). To activate AMPK, LKB1 requires two upstream kinases, STRAD and MO25 to join it in a heterotrimeric complex. This complex directly activates AMPK by phosphorylating Thr^172^ of the α subunit. The LKB1/AMPK pathway regulates the metabolic check-points of cells and stops proliferation and growth of cells in low ATP conditions. Genetic and biochemical studies in mice, worms and flies have demonstrated that LKB1 is the major phosphorylating agent of AMPK (Scott et al., 2008). Shan et al. (2016) reported that the presence of LKB1 promoted AMPK activity and its absence worked oppositely in high-fat diet-induced mice (HFD). Similarly, Hawley et al. (2003) showed that HeLa cells which were unable to express LKB1, upon exposure to external stimuli did not elevate AMPK expression. LKB1 activates AMPK in 3T3-L1 adipocytes and inhibits adipocyte differentiation (He et al., 2013). Silencing of LKB1 with siRNAs diminished the activation of AMPK in 3T3-L1 adipocytes (He et al., 2013), which pointed to the activation of AMPK by LKB1 in 3T3-L1 adipocytes. Gormand et al. (2011) have shown that LKB1 is required to maintain the normal signaling of AMPK in non-stimulated adipocytes.

Phosphorylation and activity of AMPK is also promoted by other upstream kinases with the lack of expression of LKB1 (Wang et al., 2018). Calcium acts as a trigger for AMPK activation through calcium/calmodulin-dependent protein kinase kinase-2 (also known as CaMKKβ) for phosphorylation of AMPK at Thr^172^ of the α subunit in some tissues (Desjardins and Steinberg, 2018). Unlike the LKB1 complex, CaMKKβ activates AMPK in response to an increase in the concentration of cellular Ca^2+^ regardless of changes in AMP/ADP/ATP levels (Bijland et al., 2013). Presence of Ca^2+^/CaMKK in adipocytes correspondingly regulates the activation of AMPK (Gormand et al., 2011). Chen et al. (2012) have shown the activation of AMPK and inhibition of 3T3-L1 pre-adipocyte differentiation by CaMKKβ activation. Activation of CaMKKβ reduced the expression of key adipogenic factors C/EBPα, PPARγ and SREBP-1 and activated (phosphorylated) AMPK (p-AMPK). Similarly, Lin et al. (2011) showed the inhibitory effects of CaMMKβ on adipocyte differentiation through AMPK activation. Differentiation of pre-adipocytes was enhanced in a condition of acute inhibition or deletion of CaMKKβ affirming the AMPK activation by CaMKKβ in adipocytes. Likewise, Peng et al. (2012) reported the activation of AMPK by glucagon through CaMKKβ/AMPK pathway. Glucagon enhanced the oxidation of the fatty acid and inhibited fatty acid synthesis through phosphorylation of ACC1 at Ser^79^ and ACC2 through CaMKKβ/AMPK activation in adipocytes. Moreover, it was also observed that fasting led to phosphorylated AMPK and ACC only in CaMKK^+/+^ adipocytes but not in CaMKK^–/–^ adipocytes. This demonstrates that CaMKKβ/AMPK may be the only pathway through which glucagon regulates lipid metabolism in adipocytes (Peng et al., 2012). The third upstream kinase of AMPK, TAK1 activates AMPK-α subunit (Momcilovic et al., 2006; Xiao et al., 2011; Chen et al., 2013; Wang et al., 2018). It mediates autophagy induced by tumor necrosis factor-related apoptosis-inducing ligand (TRAIL) in cancerous cells (Herrero-Martin et al., 2009). Although AMPK is phosphorylated and activated by TAK1 in different tissues and organs, LKB1 and CaMMKβ are considered the main upstream kinases of AMPK in adipocytes (Ceddia, 2013).

In the case of obesity, AMPK remains inactive due to the availability of excess nutrients and energy sources, therefore an external stimulus would be needed to activate AMPK. Much work has been performed to delineate the exogenous activators of AMPK (Coughlan et al., 2014; Goodman et al., 2014; Kim et al., 2016) and the debate is still ongoing.

### Exogenous Activators of AMPK

In recent years, much effort has been made to delineate the pathways of AMPK and to identify both direct and indirect activators (Table 1) of AMPK for the development of new therapies for various disorders including obesity. Many pharmacological and natural exogenous activators have been reported to activate AMPK either directly independent of upstream kinases or indirectly through upstream kinases.

**TABLE 1:** Some of the direct and indirect activators of AMPK.

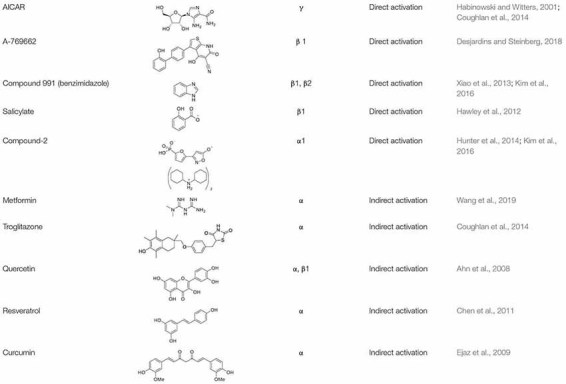

#### Direct Exogenous Activators

Activators that bind directly to AMPK and activate it without significant changes in ATP:AMP ratio are known as direct activators. Direct activators induce conformational changes in the AMPK complex, more specifically by interacting with one of the AMPK subunits. 5-Amino-4-imidazolecarboxamide riboside (AICAR) was the first identified direct activator of AMPK *in vitro* and *in vivo* (Fogarty and Hardie, 2010). AICAR has been widely used to evaluate the downstream effects of activated AMPK in animals (Fogarty and Hardie, 2010). Structurally, AICAR is similar to adenosine and it is similarly phosphorylated upon entering the cell (via adenosine transporters) to AICAR monophosphate (ZMP) by adenosine kinase. ZMP is an analog of adenosine monophosphate (AMP) and similarly activates AMPK allosterically by binding to its γ subunit. This causes an increase in Thr^172^ phosphorylation of α subunit of AMPK (Kim et al., 2016) and also inhibits the dephosphorylation of AMPK (Fogarty and Hardie, 2010). Treatment with AICAR has been shown to increase glucose tolerance, reduce TGs and free fatty acids (FFAs) level of plasma. AMPK activation by AICAR has been reported to suppress the activation of adipogenic transcription factors C/EBPα and PPARγ, and the enzymes ACC1 and FAS (Habinowski and Witters, 2001). Prolonged treatment of AMPK with AICAR increases BAT- specific protein expression; UCP1 and induces browning of WAT, thus enhances brown adipogenesis (Vila-Bedmar et al., 2010). Although AICAR has these potentially useful effects, it also has other AMPK-independent effects which limit its further use (Fogarty and Hardie, 2010). For instance, it acts on other AMP-regulated enzymes such as fructose-1,6-bisphosphatase (FBPase) and stimulates muscle glycogen phosphorylase (Longnus et al., 2003; Fogarty and Hardie, 2010). In addition, due to short half-life and poor bioavailability, it is unlikely to be used in the treatment of patients (Coughlan et al., 2014).

Other direct activators of AMPK include A-769662 compound (Thienopyridone Family), Compound 991 (Benzimidazole family), and salicylate. A-769662 is a small organic compound which activates AMPK allosterically at Ser^108^ in the AMPKβ1 subunit (Kim et al., 2016) and inhibits dephosphorylation of Thr^172^ in AMPKα subunit (Sanders et al., 2007; Goodman et al., 2014). A-769662 activates AMPK in human primary subcutaneous adipocytes (Kopietz et al., 2018) and induces thermogenesis and browning of inguinal WAT through AMPK signaling (Desjardins and Steinberg, 2018; Wu et al., 2018). Another direct activator of AMPK is Compound 991 which is reported to bind the β unit of AMPK and is more effective (5–10 fold) than A-769662 in the allosteric activation and inhibition of dephosphorylation of AMPK (Xiao et al., 2013; Kim et al., 2016). Neither A-769662 nor Compound 991 activate AMPK complexes which contain mutations in the Ser^108^ of the β subunit of AMPK, suggesting that both A-769662 and Compound 991 have a similar mechanism for the activation of AMPK (Xiao et al., 2013). Likewise, salicylate, a phytochemical obtained from willow bark (Coughlan et al., 2014) and now used very widely in the acetylated form (Aspirin) (Goodman et al., 2014), is also known to activate AMPK. Salicylate is known to directly activate AMPK in muscles, liver and WAT (Hawley et al., 2012; van Dam et al., 2015b). It binds to the β1 subunit of AMPK and activates AMPK allosterically, inhibiting the dephosphorylation of Thr^172^ in the α subunit (Hawley et al., 2012). Beyond these examples, 5-(5-hydroxyl-isoxazol-3-yl)-furan-2- phosphonic acid, termed as Compound-2 (C-2) is the most potent direct activators of AMPK. C-2 binds to the AMPKα subunit, causes allosteric activation of AMPK and prevents the dephosphorylation of Thr^172^. C-2 mimics AMP’s effects in the activation of AMPK, but unlike AICAR, it does not have any effect on the enzymes which use AMP as a substrate (Hunter et al., 2014). C-2 shows potency twice that of AMP, and 20 times that of A-769662 (Kim et al., 2016).

#### Indirect Exogenous Activators

Studies have shown that modulators which cause calcium (Ca^2+^) or AMP accumulation in the body can result in the activation of AMPK (Kim et al., 2016) without any direct interaction. These modulators are known as indirect activators of AMPK and may be physiological, pharmacological, or natural product activators (Coughlan et al., 2014; Kim et al., 2016). Pharmacological and phytochemical compounds such as metformin, troglitazone, quercetin, genistein, epigallocatechin gallate, resveratrol, berberine, curcumin and the α-lipoic acid act as indirect activators of AMPK (Kim et al., 2016), activating the kinase by the expenditure of energy because when ATP is decreased, AMP is increased. Metformin is a biguanide which is found in *Galega officinalis* (Fogarty and Hardie, 2010). It upregulates the activity of AMPK, increases the oxidation of fatty acids, downregulates lipogenic genes, increases glucose uptake and decreases the production of glucose. Metformin activates AMPK indirectly, by binding and inhibiting the complex I of the mitochondrial respiratory chain, thus increasing the AMP:ATP ratio. It also inhibits the dephosphorylation of AMPK and increases the phosphorylation of AMPK through upstream kinase of AMPK, LKB1 (Goodman et al., 2014). Metformin mediated activation of AMPK results in improved mitochondrial respiration and hyperglycemia in obesity. Recently, Wang et al. (2019) have shown the activation of AMPK by metformin in hepatocytes. Metformin mediated AMPK activation promoted mitochondrial fission, improved mitochondrial respiration and restored the mitochondrial life cycle. Thiazolidinediones (TZDs) are insulin-sensitizing drugs and consist of rosiglitazone, pioglitazone and troglitazone (Coughlan et al., 2014); these compounds indirectly activate AMPK and promote phosphorylation of ACC1 in various types of tissues including adipose, skeletal muscles and liver (LeBrasseur et al., 2006; Coughlan et al., 2014). Troglitazone caused phosphorylation and activation of AMPK in adipose tissue just after 15 min of administration (LeBrasseur et al., 2006). Similarly, pioglitazone also caused rapid phosphorylation of AMPK and ACC1 in Swiss 3T3-fibroblast cells (LeBrasseur et al., 2006). TZDs enhance the accumulation of AMP by inhibiting the complex I of the mitochondrial respiratory chain and hence activate AMPK indirectly (Brunmair et al., 2004). Moreover, they enhance the expression of PPARγ which in turn increases the release and expression of adiponectin from adipocytes (LeBrasseur et al., 2006), which activates AMPK in skeletal muscle and liver, increases the oxidation of fatty acids and uptake of glucose, and decreases the production of hepatic glucose (Kim et al., 2016).

Indirect activation of AMPK by phytochemicals has also been reported in numerous studies. Quercetin is one of the most abundant flavonoids found in many plants, food and grains, and is known to activate AMPK indirectly (Ahn et al., 2008). Exposure of 3T3-L1 cells to quercetin resulted in decreased expression of positive regulators of adipogenesis, and indeed attenuation of adipogenesis. This was due to the phosphorylation of AMPK α and β1 subunits, and its downstream substrate ACC (Ahn et al., 2008). Another indirect activator of AMPK that can be found in grapes is resveratrol. Resveratrol activates AMPK α indirectly by increasing AMP:ATP ratio through inhibition of mitochondrial ATP production (Fogarty and Hardie, 2010; Hawley et al., 2010; Chen et al., 2011). Resveratrol activates AMPK and enhances brown adipogenesis (Wang et al., 2015). Treatment with resveratrol has been shown to stimulate mitochondrial biogenesis, glucose uptake and reduce the accumulation of lipids in different types of cells (Baur et al., 2006; Zang et al., 2006; Um et al., 2010; Coughlan et al., 2014). Wang et al. (2015) observed enhanced mRNA and protein expression of brown adipocytes-specific markers such as UCP1, PRDM16, PGC-1α etc, in inguinal WAT after treatment with resveratrol. It was observed that the formation of brown-like adipocyte formation in WAT was through activation of AMPK α1. Such brown adipocyte formation in WAT was absent in cells lacking AMPK α1, which demonstrates the positive role of AMPK in brown adipogenesis and browning of WAT. In addition, curcumin derived from *Curcuma longa* also activates AMPK through its α subunit. Exposure of 3T3-L1 adipocytes to curcumin enhanced the phosphorylation and activation of AMPK and decreased the expression of ACC by phosphorylation (Ejaz et al., 2009).

Unsurprisingly, physiological activators, for instance, exercise and calorie restriction, induce the increase in AMP:ATP and indirectly activate AMPK. Contraction of muscles both in human and rodents results in activated AMPK, giving one of the most compelling examples of molecular effects of exercise (Chen et al., 2003; Coughlan et al., 2014). While intracellular energy level is a crucial determinant in the activity of AMPK, it has been reported that reactive oxygen species (ROS) also induced the activation of AMPK without any decrease in ATP level (Quintero et al., 2006; Wu et al., 2012; Kim et al., 2016).

## Summary and Outlook

Obesity is a common disorder caused by the interaction of environmental, genetic and nutritional factors, and its pervasiveness is accelerating worldwide. Socioeconomic changes, extensive consumption of calorific foods, and increasingly sedentary lifestyles are the predominant causative factors for abnormal adipose tissue development and rise in obesity. Abnormalities, both in the development of adipose tissue and the differentiation of pre-adipocytes to mature adipocytes are directly linked to obesity. Adipose tissue has a strong influence on whole-body metabolism and therefore is an attractive site for anti-obesogenic therapies. A cascade of hundreds of transcriptional factors and signaling pathways act as either negative or positive modulators of adipose tissue development and adipogenesis. Significant efforts had been made in the past few years to gain insight into the molecular modulation of adipogenesis, but the investigation into promising targets and identification of unique regulators of adipogenesis including signaling pathways are still elusive and needed in the fight against obesity. The heterogeneity of adipose tissue increases the challenges of determination of the exact role of various signaling intermediates and AMPK in different depots. The control of white adipogenesis accompanied by reduction of lipid contents in mature white adipocytes, numeric decrease of adipocytes and controlling the abnormal production of cytokines (adipokines) can be an effective strategy to combat obesity. Moreover, activation of AMPK in adipose tissue could prove beneficial in attenuating adipose tissue dysfunctionality because AMPK has a crucial role in the regulation of transcriptional factors and pathways related to white/brown adipogenesis and lipid synthesis. AMPK activation could also prove beneficial in the prevention of various other pathological conditions associated with obesity such as type-2 diabetes, cancer, chronic inflammation etc. Obesity is directly linked to chronic inflammation, and chronic inflammation is a risk factor of many diseases. Thus inhibition of adipose-derived pro-inflammatory cytokines through activation of AMPK could help in the attenuation of metabolic syndrome. In addition, the role of AMPK, especially in BAT, must be investigated in detail, as brown adipogenesis is inversely proportional to obesity and associated complications. AMPK, therefore, can thus be a potential therapeutic target in the prevention and treatment of obesity and we believe that these steps could expedite the development of anti-obesogenic drugs against obesity.

## Author Contributions

BA and EW performed the literature search, designed and wrote the draft of the manuscript. CS and IF edited the manuscript and contributed to structure and composition. All the authors reviewed and approved the final version of the manuscript before submission.

## Conflict of Interest

The authors declare that the research was conducted in the absence of any commercial or financial relationships that could be construed as a potential conflict of interest.
